# Evidence for photo-induced charge separation between dye molecules adsorbed to aluminium oxide surfaces

**DOI:** 10.1038/srep21276

**Published:** 2016-02-19

**Authors:** Ute B. Cappel, Davide Moia, Annalisa Bruno, Valerie Vaissier, Saif A. Haque, Piers R. F. Barnes

**Affiliations:** 1 Department of Chemistry, Imperial College London, SW7 2AZ, UK; 2 Department of Physics, Imperial College London, SW7 2AZ, UK; 3 Department of Chemistry, Massachusetts Institute of Technology, 77 Massachusetts avenue, Cambridge, MA 02139, USA; 4 Italian National Agency for New Technologies, Energy and Sustainable Economic Development (ENEA), Portici (Naples), Italy

## Abstract

Excited state dynamics and photo-induced charge transfer of dye molecules have been widely studied due to their relevance for organic and dye-sensitised solar cells. Herein, we present a femtosecond transient absorption spectroscopy study of the indolene dye D131 when adsorbed to inert Al_2_O_3_ substrates for different surface concentration of the dye. Surprisingly, we find that at high surface concentrations, the first singlet excited state of the dye is converted into a new state with an efficiency of about 80%. We assign the absorption features of this state to the oxidised dye and discuss the possibility of photo-induced charge separation between neighboring dye molecules. Our study is the first to show that this process can be highly efficient without the use of donor and acceptor molecules of different chemical structures.

Photo-induced charge transfer between molecules is of interest in a variety of research fields, such as dye-sensitised solar cells^1^,^2^, organic solar cells^3^,^4^,^5^ and artificial photosynthesis^6^,^7^,^8^. In such a process, charge separation usually occurs when the binding energy of the excited state is overcome by an electron (hole) being transferred to a lower (higher) energy level. Therefore, this charge transfer reaction involves acceptors and donors of different chemical structures. Both intra- and intermolecular charge transfer are possible depending on whether the acceptor is chemically linked to the donor. Examples of such transfer processes include: exciton separation between donor and acceptor molecule interfaces in organic solar cells, electron injection from dye molecules into semiconductor acceptor states, dye regeneration by molecular hole conductors in dye-sensitised solar cells and charge separation in photosynthesis.

In this communication, we consider photo-induced inter-molecular charge transfer between dye molecules of the same chemical structure adsorbed to an inert aluminium oxide substrate. Al_2_O_3_ has been widely used as an inert reference material in DSC studies due to the high energy of its conduction band^9^, which prevents electron injection from excited dyes. Therefore, Al_2_O_3_ has been used to study the excited state dynamics of dye molecules when bound to a metal oxide surface and to calculate electron injection yields into non-inert substrates such as TiO_2_ by comparison of the excited state lifetimes. We demonstrate that a charge separation process might occur between dye molecules when they are adsorbed to an Al_2_O_3_ surface and that the charge transfer can be inhibited by controlling the packing of molecules on the surface through use of a co-adsorber that separates the dyes.

## Results

**Figure 1:**
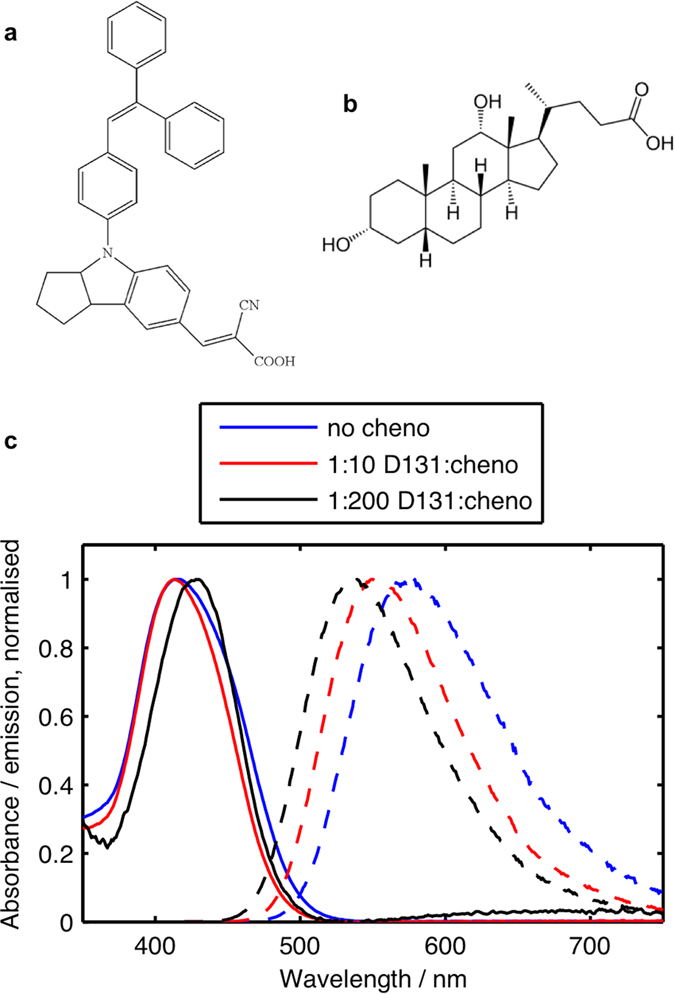
Chemical structures of (**a**) D131 and (**b**) deoxycholic acid (cheno). (**c**) Normalised absorption (solid lines) and emission (dashed lines) spectra of D131 on Al_2_O_3_ with different amounts of cheno added in the dye bath.

The indolene dye D131^10^,^11^ (Fig. 1a) and the co-adsorber deoxycholic acid^12^,^13^ (cheno, Fig. 1b) were used in this study. When preparing films, the concentration of the dye in the solutions used for sensitisation was kept constant while increasing amounts of cheno were added. Fig. 1c shows the normalised absorption and emission spectra of D131 adsorbed to 400 nm thick mesoporous Al_2_O_3_ films sensitised without cheno, 10 times the concentration of cheno in the dye bath and 200 times the concentration of cheno in the dye bath compared to the dye. Table 1 summarises the absorption and emission parameters of the same samples. Upon addition of cheno to the dye bath, small changes in the absorption spectra of films are observed. With the lower concentration of cheno, the absorption spectrum becomes slightly narrower compared to the spectrum without cheno and with the higher cheno concentration it also red-shifts. In contrast, the emission spectra are increasingly blue-shifted with the addition of cheno leading to a smaller Stokes shift. The absorbance of the samples decreased with increasing cheno concentration leading to a reduction of the dye concentration on the surface to 60% for 1:10 dye:cheno and to 12% for 1:200 dye:cheno compared to the concentration without cheno (Table 1 and Supplementary Fig. 1). In the remainder of this communication, we will therefore refer to the samples as 100%, 60% and 12% dye coverage, respectively. In contrast to the absorbance, the emission increased by a factor of 4 for 1:10 dye:cheno and by a factor of 8 for 1:200 dye:cheno compared to the sample without cheno (Table 1 and Supplementary Fig. 2). The emission intensity was evaluated by integrating the emission spectra and scaling for the absorptance (fraction of absorbed photons) at the excitation wavelength of 400 nm. The absorption and emission data therefore suggests that the addition of cheno to the dye bath was successful in reducing Al_2_O_3_ surface coverage and dye-dye interactions on the surface. Given the small changes in the absorption data, there is no evidence to suggest the formation of ordered H- or J-type aggregates on the surface in absence of cheno in the dye bath. For H-aggregates a more pronounced red shift of the absorption spectra and a much stronger fluorescence quenching in absence of cheno would be expected than is observed^14^. In the case of J-aggregates, the formation of aggregates would be expected to lead to a strong red-shift of the absorption compared to the monomer, which is not observed here.

**Figure 2:**
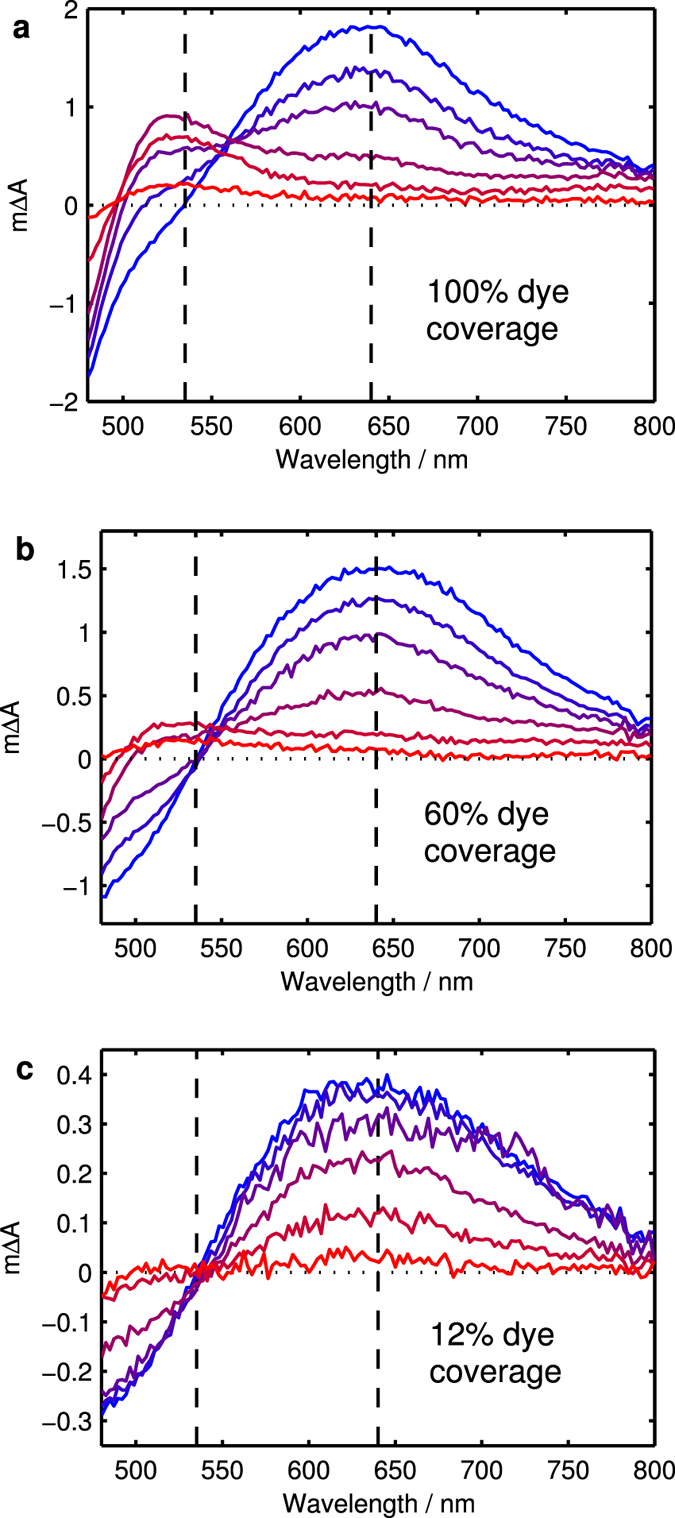
TA spectra at time delays of 0.5 ps, 1.5 ps, 5 ps, 50 ps, 500 ps and 5000 ps going from blue to red for (**a**) D131 on Al_2_O_3_, (**b**) D131 on Al_2_O_3_ with 1:10 dye to deoxycholic acid in dye bath, (**c**) D131 on Al_2_O_3_ with 1:200 dye to deoxycholic acid in dye bath excited at 400 nm with 25 μJ cm^−2^. The last spectrum of (**c**) was at 3000 ps instead of 5000 ps.

**Figure 3:**
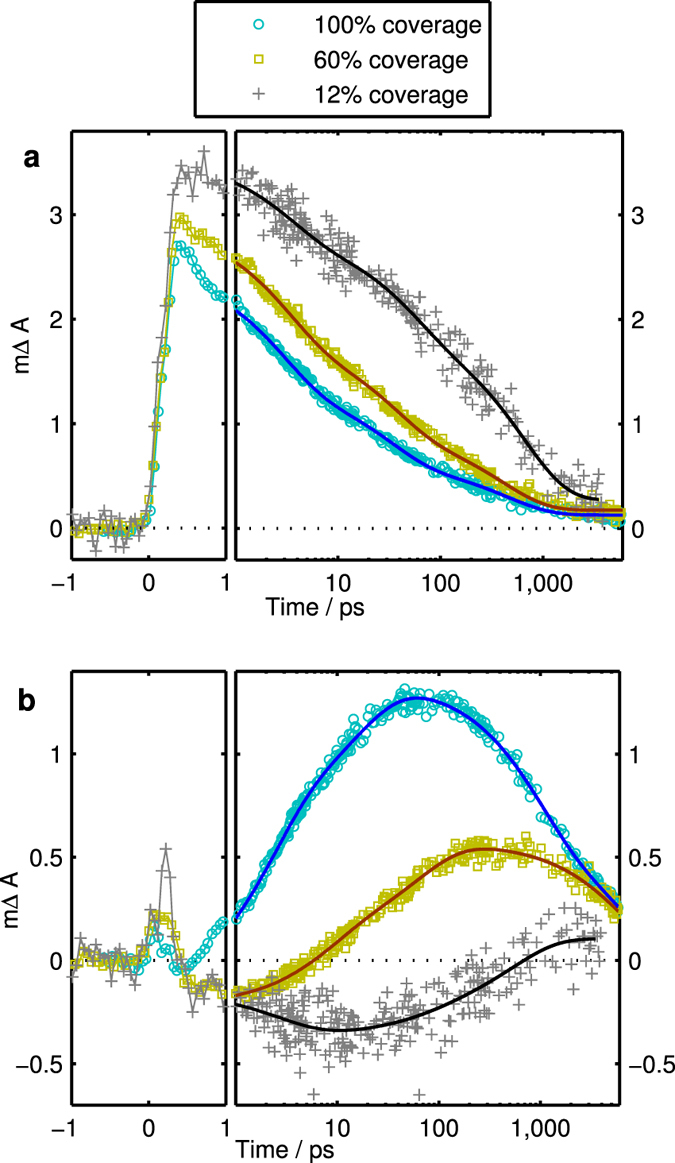
TA kinetics at (**a**) 640 nm and (**b**) 535 nm for D131 on Al_2_O_3_ without deoxycholic acid (100% dye coverage, blue dots), with 1:10 D131:deoxycholic acid in the dye bath (60% dye coverage, yellow squares) and with 1:200 D131:deoxycholic acid in the dye bath (12% dye coverage, grey crosses). Solid lines from 1 ps onwards represent multi-exponential fits to the data.

We assessed the charge formation and dynamic behaviour of the samples upon photo-excitation using femtosecond transient absorption spectroscopy (TAS). Samples were excited with a femtosecond laser pulse at 400 nm and TA spectra were measured at different pump-probe delay times. Selected TA spectra for the three samples are shown in Fig. 2. At short delay times (0.5 ps), the spectra of all samples show a TA peak at 640 nm and a bleach at wavelengths shorter than 535 nm (these wavelengths are indicated by black dashed lines in Fig. 2). This spectral signature is assigned to the excited state of D131 in agreement with the spectral features for a sample of D131 dispersed in polystyrene (Supplementary Fig. 3) and results in literature^15^. In the sample without cheno (100%), the features of the excited state decrease with time while simultaneously a new peak emerges at 520 nm (Fig. 2a). This new peak is also observed for 60% dye coverage but its magnitude relative to the excited state peak is much smaller. In contrast, this peak is not observed for 12% dye coverage and was not observed for D131 in polystyrene either. In order to follow the excited state decay and the formation of the new state, kinetic traces at 640 nm and 535 nm normalised to the absorptance at the excitation wavelength are shown in Fig. 3. The kinetic data were fitted with multi-exponential decays according to Equation 1 from 1 ps onwards (see Supplementary Table 1) and average time constants of these fits calculated using Equation 2 (Table 2).


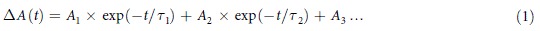



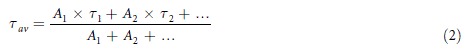


Figure 3a shows that the initial excited state amplitudes increase with decreasing dye coverage and that the excited state decay is slower. The average excited state lifetime increases by a factor of 3 for 12% coverage compared to 60% coverage (Table 2). A similar trend has also been observed in femtosecond upconversion emission data (Supplementary Fig. 4) collected at 580 nm where it is clear that the fluorescence life time increases by a factor of four from 100% to 12% surface coverage (Supplementary Table 2). Samples were excited with a 100-fs laser pulse at 400 nm and details of the setup are reported in a previous paper^16^.

The kinetic traces at 535 nm clearly show the rise of the new peak for 100% coverage and for 60% coverage (Fig. 3b). For 12% coverage, the kinetics mostly seem to reflect a small change in the shape of the excited state spectrum but no clear rise of the signal to positive values was observed. Therefore, while the results of a multi-exponential fit to the data are included in Fig. 3b as a guide to the eye, the fit results are not included in Table 2. For the other two samples, the clear rise of the new peak was followed by a decay of this peak and average time constants for both the rise and the decay are summarised in Table 2. The rise time constants are shorter than excited state decay times suggesting that not all excited states are converted into the new state. The rise is slower for 60% coverage and the maximum amplitude of the peak at 520 nm is reduced by more than a factor of two. The conversion into the new state therefore appears less efficient with 60% dye coverage compared to 100% dye coverage. The lifetimes of the new state are in the order of a few nanoseconds and therefore about an order of magnitude longer than the lifetimes of the excited state in the absence of the conversion process (for 12% coverage).

## Discussion

We now turn to the reason for the appearance of this new state. It has been shown that some dyes can inject electrons into surface states of large band gap metal oxides for ZrO_2_^17^,^18^,^19^ and even for Al_2_O_3_^20^. Furthermore, it has been shown that indolene dyes can photo-isomerise in solution leading to changes in the transient absorption spectra^21^,^22^. However, in our case the appearance of the new state is dependent on the surface concentration of the dye and its appearance can be inhibited when the dye coverage becomes low. This suggests that neither an interaction with the substrate nor a process occurring in individual dye molecules is responsible for the appearance of the new state.

D131 is expected to pack tightly on oxide surfaces and its binding geometry on TiO_2_ has been predicted to make the dye stand up^23^. Furthermore, it has been shown that hole transfer between adjacent dye molecules is possible^24^. Therefore, it is likely that emergence of the new state stems from an interaction of dye molecules on the surface. While it is likely that excited states can be transferred from one dye molecule to another when dye molecules are closely packed, this process is not expected to lead to a significant change of shape of the excited state spectrum. The new state therefore has to be due to an energy or electron transfer process, which has a significantly different absorption spectrum to the excited state. Triplet formation is unlikely to occur on the observed timescales in an all-organic dye like D131. Furthermore, we excluded oxygen during the transient absorption measurements and triplet states should therefore be much longer lived than the lifetimes of a few nanoseconds observed for the new states here. A further consideration is whether the new state could be due to excimer formation, i.e. the association of two neighbouring molecules after one of them is excited. Formation of excimers has been shown to occur within a few picoseconds for covalently perylenediimide dimers in solution^25^,^26^. An excimer should show a red-shifted fluorescence band compared to the monomer as the excimer should be a lower energy state than the singlet excited state. While we see a red-shift of the fluorescence with increased dye surface coverage, this is manifested in a continuous shifting from low to high coverage rather than in the emergence of a new emission band. Furthermore, our observed fluorescence lifetimes are correlated to the initial excited state rather than to the new state formed. We therefore suggest that emission only occurs from the initial excited state and that the new state formed is due to a charge transfer process.

**Figure 4:**
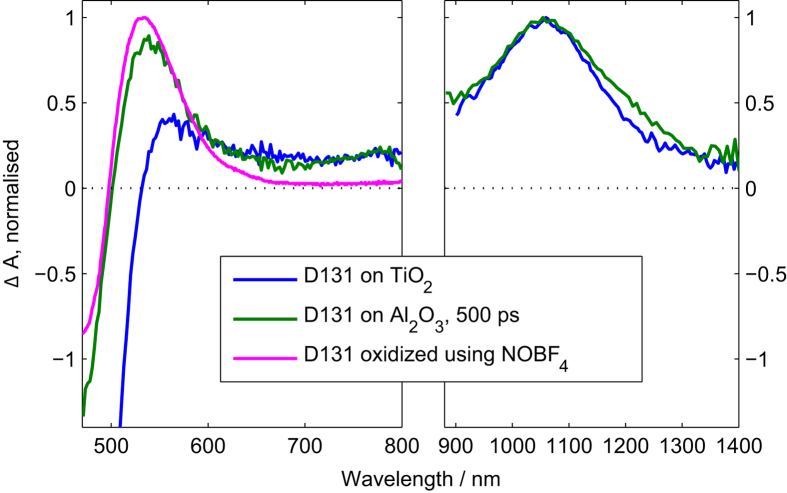
TA spectra of D131 on TiO_2_ and Al_2_O_3_ (100% coverage), 500 ps after excitation with a 450 nm pump, normalised at 1050 nm. On the left the difference in absorbance of a D131 solution in dichloromethane after and before partial oxidation with NOBF4 (0.5:1 ratio of oxidant to D131) is overlaid (magenta line).

Additional TA measurements of D31 on Al_2_O_3_ (100% coverage) in the near infrared region show the conversion of the excited D131 peak at 1250 nm into a peak centred at 1050 nm (Supplementary Fig. 5). A comparison of the extended TA spectrum 500 ps after excitation with a TA spectrum of D131 absorbed to TiO_2_ reveals that both spectra show the peak at 1050 nm (Fig. 4). This peak has been assigned to the oxidised from of D131, in agreement with efficient electron injection into TiO_2_ 27. Furthermore, the D131 spectrum on TiO_2_ shows the shoulder of a peak at 550 nm which seems to be overlaid with the negative features arising from a Stark shift of the dye’s ground state absorption caused by electrons in TiO_2_ 28. Chemical oxidation of D131 in solution using NOBF_4_ also shows a peak at approximately 530 nm (Fig. 4 and Supplementary Fig. S6). For D131 on Al_2_O_3_ the peak at 535 nm and the peak at 1050 nm decay with the same time constants (Supplementary Fig. 5), confirming that they arise from the same species. We therefore assign the new features observed for D131 on Al_2_O_3_ to oxidised D131. This means that excited D131 is converted to oxidised D131 for high surface coverages on Al_2_O_3_. Using the steady-state PL intensities and assuming that conversion does not occur at 12% coverage and that all loss in PL intensity at higher coverages is due to conversion to the new state, we can estimate conversion efficiencies from the PL intensity. This estimation leads to a conversion efficiency of 88% for 100% coverage and a conversion efficiency of 50% for 60% coverage. The conversion efficiency can also be determined from the maximum amplitude of the TAS data at 535 nm using the extinction coefficient of D131^+^ at 535 nm (determined to be 19,400 M^−1^ cm^−1^ using spectroelectrochemistry, Supplementary Fig. S7). From this calculation, 77% of all absorbed photons at 100% coverage and 33% of all absorbed photons at 60% coverage are converted to the oxidised dye. These values are somewhat lower than the values estimated from PL but still in good agreement. Both methods of estimation suggest very high conversion efficiencies for high surface coverage and give a further indication that the conversion is not due to injection into surface states of Al_2_O_3_, as electron injection into surface states of high conduction band metal oxides has been typically associated with very low injection efficiencies.

We also measured the wavelength and excitation intensity dependence of the conversion for 100% surface coverage on Al_2_O_3_ (Supplementary Fig. S8 and S9). The efficiency of conversion decreases somewhat with increasing wavelength but the conversion still occurs over a similar time range even for excitation close to the band gap. This suggests that excitation to a higher lying state than the first singlet excited state is not necessary for the conversion and that the process can occur after thermalisation of the excited state. The conversion shows almost no excitation intensity dependence except for becoming slightly less efficient at very high excitation intensities. This indicates that the process is a single excited state process and not due to exciton-exciton annihilation^29^,^30^,^31^.

**Figure 5:**
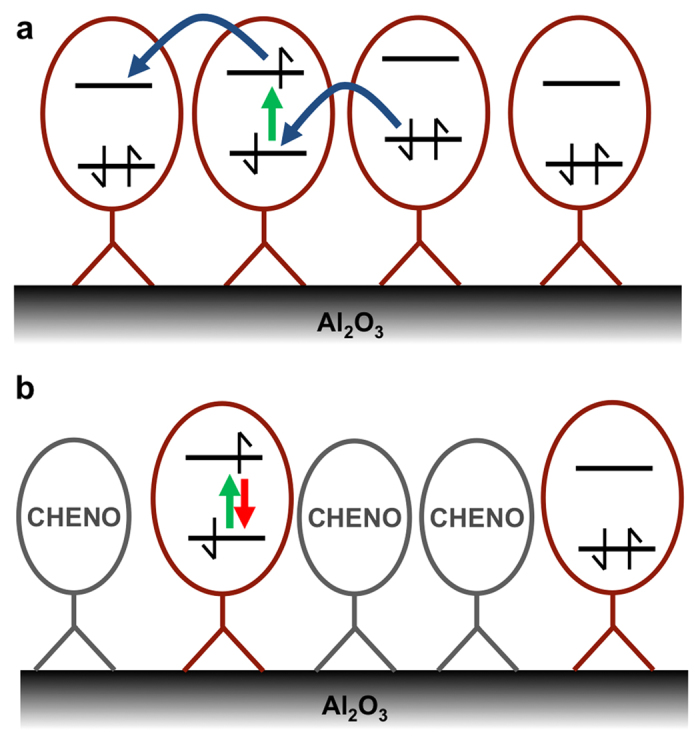
Schematic diagram of D131 molecules on Al_2_O_3_ surface. (**a**) High coverage: Molecules are closely packed and energetic disorder allows for either photo-induced electron or hole transfer from an excited molecule to a neighbouring molecule leading to an oxidised and a reduced molecule on the surface. (**b**) Low coverage in the presence of deoxycholic acid: Photo-induced charge transfer is inhibited.

We therefore suggest that emergence of the oxidised dye on the surface is due to photo-induced electron or hole transfer from an excited dye molecule to one of its neighbours, resulting in the formation of an oxidised and a reduced dye molecule on the surface (Fig. 5a). While we clearly observe absorption features due the oxidised dye in transient absorption spectroscopy, we do not observe any specific features of the reduced dye. We were unable to measure the absorption spectrum of the reduced dye experimentally, and therefore we used TDDFT calculations to predict its absorption spectrum (Supplementary Fig. 10). These calculations suggest that the main absorption peak of the reduced dye overlaps with the absorption of the ground state and the oxidised dye. As we cannot observe the reduced dye independently as its concentration will always be equal to the concentration of the oxidised dye, this spectral overlap prevents us from observing it. A further absorption peak overlaps with the oxidised absorption in the near infrared but has a comparatively weak oscillator strength. Therefore, we do not expect specific features of the reduced dye in transient absorption spectra. Addition of cheno and reduction of the surface coverage of the dye will inhibit a dye-dye charge transfer process as such a process will only be efficient at short dye-dye distances (Fig. 5b). A further requirement is a driving force for the charge separation process in order to overcome the exciton binding energy. While there is no overall driving force for this reaction, since the charge separation occurs between identical molecules, local energetic differences between neighbouring dyes might be sufficient to drive the transfer. We then performed two types of calculations to assess the feasibility of charge separation in our system.

First, we applied quantum chemical methods to calculate the energy of the reactants (one excited and one ground-state D131 molecule) and the products (one oxidised and one reduced D131 molecule) of the charge transfer reaction. Since the products are two charged molecules, we needed to account for the electrostatic interaction between them. To this end, we used charge constrained Density Functional Theory (DFT) on a pair of dyes separated by 8 Ångströms, while we calculated the energy of the ground and excited state reactant molecules with DFT and Restricted Open Kohn Sham (ROKS) respectively. We found an energy difference of 0.25 eV (Supplementary Fig. 11).

Second, we predicted the likely maximum energy differences between neighbouring dye molecules (Supplementary Fig. 12). Variations in the energy landscape of dye molecules on TiO_2_ surfaces have been discussed previously and predicted to have a Gaussian distribution with a full width at half maximum of 0.4 eV 32. Assuming a similar distribution of energies here and a 6 nearest neighbours configuration, we estimate that 80% of dyes have an energy difference of 0.26 eV or more to at least one of its neighbours. It has to be noted that fewer molecules exhibit this energy difference if the number of nearest neighbour is reduced. On the other hand, if exciton diffusion occurs prior to charge separation, the likelihood of reaching a site with an even larger energy difference is increased.

In summary, the energy variations in the dye monolayer adsorbed at the surface of the metal oxide seem to be of sufficient magnitude to overcome the energy difference of the charge separation reaction. Therefore, we demonstrated that exciton splitting in our system can result in a charge transfer state across two neighbouring dye molecules.

Polaron formation from excitons has been previously observed in films of conjugated polymers^33^,^34^,^35^ achieving up to 30% efficiency^34^. For regioregular poly-3-hexylthiophene (RR-P3HT) it has been shown that polarons form from hot excitons^35^ and at interfaces between molecularly ordered and disordered domains^33^. In contrast to these previous examples our results are unique in that:

- Charge separation is highly efficient (more than 80% conversion efficiency) despite having no “in-built” driving force within the system
- Charge separation can occur from both hot and relaxed excited states
- Molecules are arranged in a 2-D structure on the surface of an insulator.

The observations presented here therefore demonstrate that when studying molecular monolayers on surfaces not only intermolecular charge transfer and exciton transfer have to be considered but that also exciton splitting/charge separation can occur.

In conclusion, we have shown that excitation of a dye on an aluminium oxide surface can lead to the conversion of the excited state to a new state. We suggest that this is due to a “self” charge separation process in which the exciton is split into an electron and a hole on adjacent dye molecules. This process leads to an increase in the lifetime of the excitation by about an order of magnitude compared to excited states, which do not separate. The charge separation can be inhibited by control of the dye’s surface concentration (and so the average distance between the dyes) through the use of a co-adsorber in the dye bath.

## Methods

### Sample preparation

Al_2_O_3_ paste was prepared as previously described.^36^ The paste was then diluted in ethanol / water and spun-coat to obtain a thickness of approximately 400 nm. Films were then sintered for 30 minutes at 450 degrees in an oven. Following this, films were dyed for 2 hours in 0.1 mM D131 (purchased from Mitsubishi paper mills limited) dissolved in a 1:1 mixture of acetonitrile and tert-butyl alcohol. Where applicable, the solutions also contained deoxycholic acid (purchased from Sigma Aldrich). A film of D131 dispersed in polystyrene (M_W_ = 250,000 g/mol) was fabricated by spin-coating a solution of 2 mg/ml D131 and 200 mg/ml polystyrene in chlorobenzene at 2000 rpm.

### Steady-state absorption and emission spectroscopy

UV-visible absorption spectra were measured on a Shimadzu 2600 spectrophotometer with an ISR-2600Plus Integrating Sphere Attachment, so that the transmittance (T) and the reflectance (R) of the films could be measured. The absorbance of the samples was calculated from −log10 T, while the absorptance was calculated from 1-T-R. Fluorescence spectra were measured with a Horiba Jobin Yvon Fluorolog-2 fluorometer.

### Femtosecond transient absorption spectroscopy

Femtosecond TAS was measured using a HELIOS spectrometer from Ultrafast Systems^37^. Pump and probe pulses were generated from the output of a Solstice Ti:Sapphire regenerative amplifier (Spectra-Physics) with 800 nm 92 fs pulses with a 1 kHz frequency. The probe pulse travelled through a delay stage and was then converted into either visible or near-infrared light using a sapphire crystal. It was then split into a signal and a reference beam and the signal beam was focussed onto the sample. The pump pulse was created using an optical parametric amplifier and a series of filters (TOPAS-NIRUVIS). It was then chopped mechanically to obtain a 500 Hz train of pulses. The pump pulse was directed onto the sample in the same spot as the probe beam but not focussed. The intensity of the pump pulse at the sample was adjusted through neutral density filters and measured through a pinhole before and after each experiment. TA spectra were calculated from the difference in detected probe light in presence and absence of the pump pulse and automatically adjusted for fluctuations in probe intensity using the reference part of the probe beam. The samples were kept under nitrogen flow in a cuvette throughout the measurements.

## Additional Information

**How to cite this article**: Cappel, U. B. *et al.* Evidence for photo-induced charge separation between dye molecules adsorbed to aluminium oxide surfaces. *Sci. Rep.* **6**, 21276; doi: 10.1038/srep21276 (2016).

## Supplementary Material

Supplementary Information

## Figures and Tables

**Table 1:** Absorption and emission parameters of D131 on Al_2_O_3_.

Ratio, D131: cheno	1:0	1:10	1:200
D131 concentration/mM	0.1	0.1	0.1
Cheno concentration/mM	0	1	20
Absorption maximum/nm	415	413	428
Fluorescence maximum/nm	575	550	538
Absorbance at maximum	0.67	0.40	0.08
Relative dye coverage/%	100	60	12
Absorptance at 400 nm/%	71	52	11
Rel. Fluorescence intensity	1	4	8

Absorbance values were calculated from −log T. Absorptance values were calculated from 1-T-R. Relative fluorescence intensities were calculated from the sum of the PL spectra corrected for the absorptance at the excitation wavelength (400 nm) relative to the sample without cheno.

**Table 2:** Average time constants obtained from tri-exponential fitting of the kinetic traces at 640 and 535 nm.

	τ_av_ at 640 nm/ps	τ_av rise_ at 535 nm/ps	τ_av decay_ at 535 nm/ps
100% coverage	100	7.2	3600
60% coverage	120	39	6700
12% coverage	330	n.a.	n.a.
