# Supplementary material for: Evidence for photo-induced charge separation between dye molecules adsorbed to aluminium oxide surfaces
Source: Sci Rep. 2016 Feb 19;6:21276. doi: 10.1038/srep21276 (PMC4759562; doi:10.1038/srep21276)
Supplement: Supplementary Information [file srep21276-s1.pdf]

## Supplementary Information for:

# Evidence for photo-induced charge separation between dye molecules adsorbed to aluminium oxide surfaces

Ute B. Cappel,[a,c]* Davide Moia,[b] Annalisa Bruno,[a,d] Valerie Vaissier,[e] Saif A. Haque,[a] Piers R. F. Barnes[b]

a Department of Chemistry, Imperial College London, SW7 2AZ, UK

b Department of Physics, Imperial College London, SW7 2AZ, UK

c Current address: Department of Physics and Astronomy, Molecular and Condensed Matter Physics, Uppsala University, Box 516, 751 20 Uppsala, Sweden.

d Current address: Energy Research Institute @ NTU (ERI@N), Research Techno Plaza, X-Frontier Block, Level 5, 50 Nanyang Drive, 637553, Singapore

e Department of Chemistry, Massachusetts Institute of Technology, 77 Massachusetts avenue, Cambridge, MA 02139, USA

*Ute.cappel@physics.uu.se

## S1 Supplementary Figures

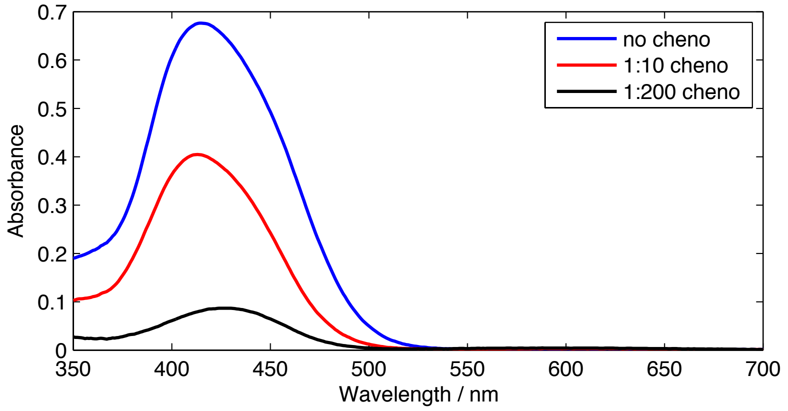


**Supplementary Figure 1:** Absorption spectra of D131 on $Al_2O_3$ with different amounts of cheno added in the dye bath.

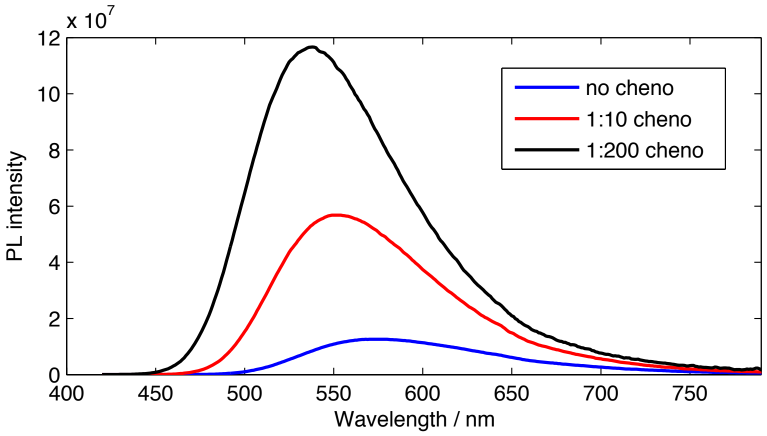


**Supplementary Figure 2:** Emission spectra of D131 on $Al_2O_3$ with different amounts of cheno added in the dye bath scaled by fraction of absorbed photons at excitation wavelength.

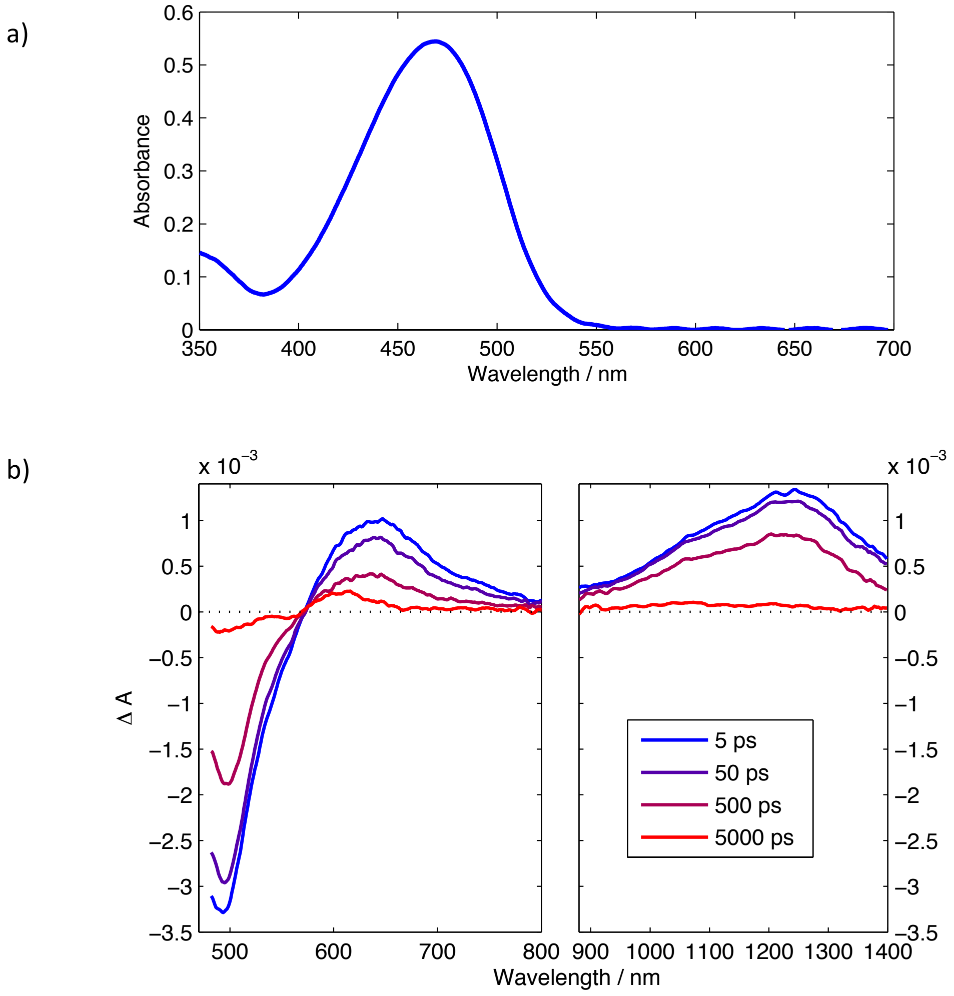


**Supplementary Figure 3:** D131 in polystyrene. a) Absorption spectrum b) Femtosecond TAS spectra of D131 dispersed in polystyrene measured with an excitation wavelength of 450 nm and 23 µJ $cm^{-2}$. The spectra show the characteristic peak of the singlet excited state of D131 at 640 nm and 1250 nm.

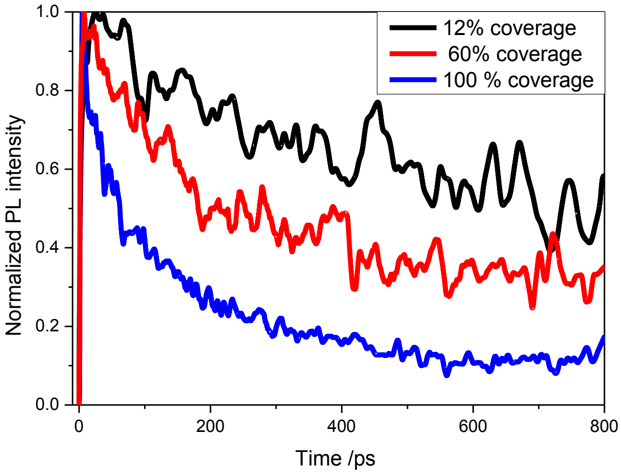


**Supplementary Figure 4:** Fluorescence upconversion kinetics of D131 on $Al_2O_3$ for different surface coverages (namely blue line 100%, redline 60 %, and black line 12 %). Excitation wavelength: 400 nm, detection wavelength: 580 nm. Fluorescence up-conversion measurements were performed using a femtosecond laser system. The Second Harmonic output of a mode-locked Ti:Sapphire oscillator with the wavelength fixed at 800 nm was used as source beam. The pulse duration was 70 fs and the repetition rate 80 MHz. A portion of this fundamental beam was frequency doubled to create the excitation beam with a wavelength of 400 nm. Fluorescence from the sample was focused on a beta barium borate (BBO) crystal along with the 800 nm fundamental beam. The fluorescence emitted at different emission wavelengths was mixed in the BBO crystal with the gate beam to generate sum frequency photons which were detected using a photomultiplier tube. Data were acquired using Lab-View software and subsequently analyzed. The temporal reconstruction of the signal was obtained through a micrometrical precise delay line on the gate beam. The resolution of the system was measured to be 150 fs. Sample degradation was avoided by performing the measurements under flowing nitrogen and using a translation stage to move the sample within the beam, removing the effect of photo bleaching and providing data averaged across the whole sample.

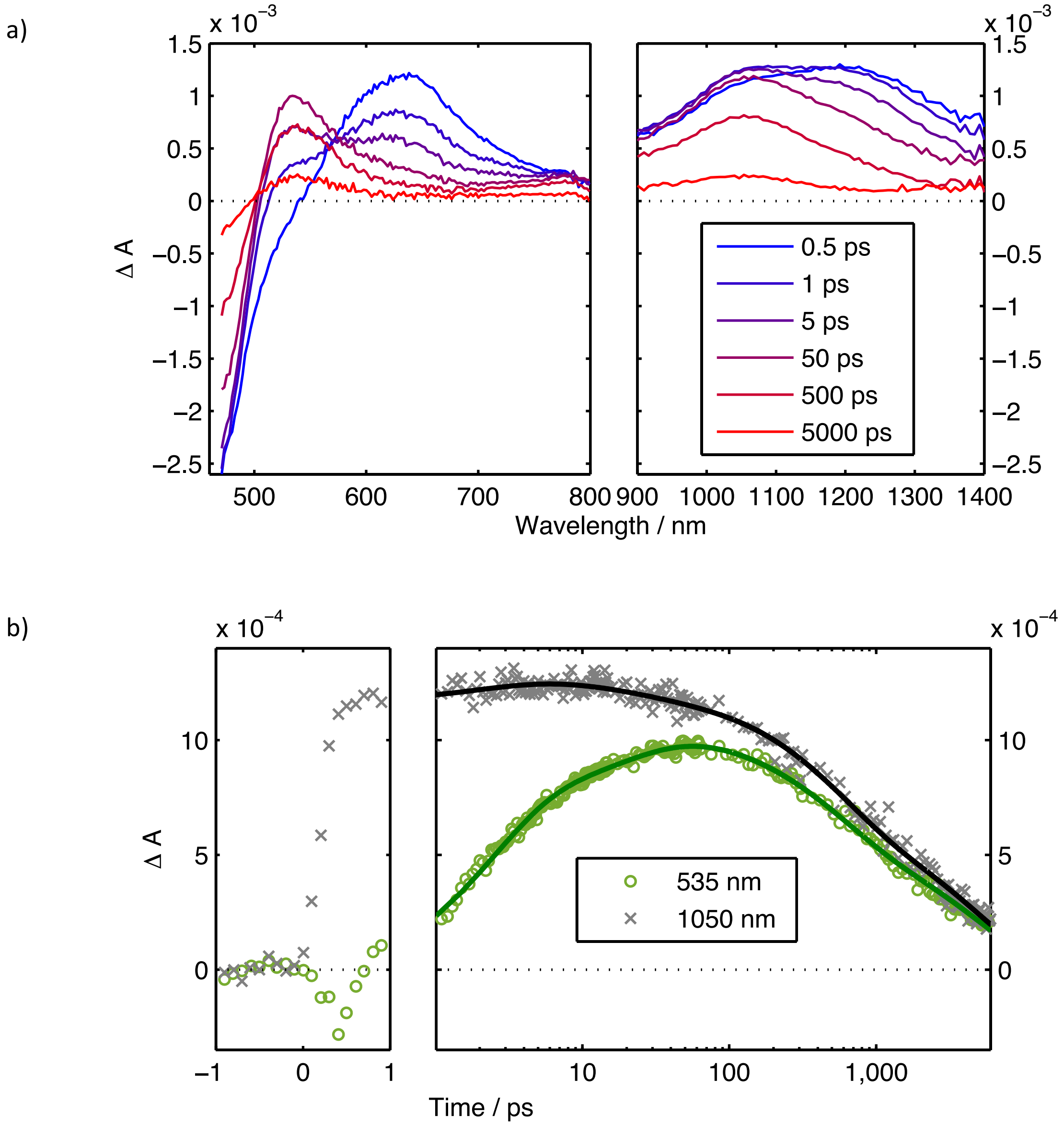


**Supplementary Figure 5:** a) Transient absorption spectra of D131 on $Al_2O_3$ (100% coverage) with 450 nm excitation. The spectra in the NIR region at short delay times show the peak of the excited state of the dye at 1250 nm, which spectrally overlaps with the peak of the oxidised dye at 1050 nm, which is observed at longer delay times. b) Kinetic traces at 535 nm and 1050 nm following the evolution of the oxidised dye signal. Multiple exponential fits between 1 and 6000 ps are shown as solid lines. The same time constants were used in fitting both curves demonstrating that the same processes are responsible for the formation and the decay of the signal. At 1050 nm, no clear rise of the signal is observed as the both the oxidised and the excited dye absorb at this wavelength.

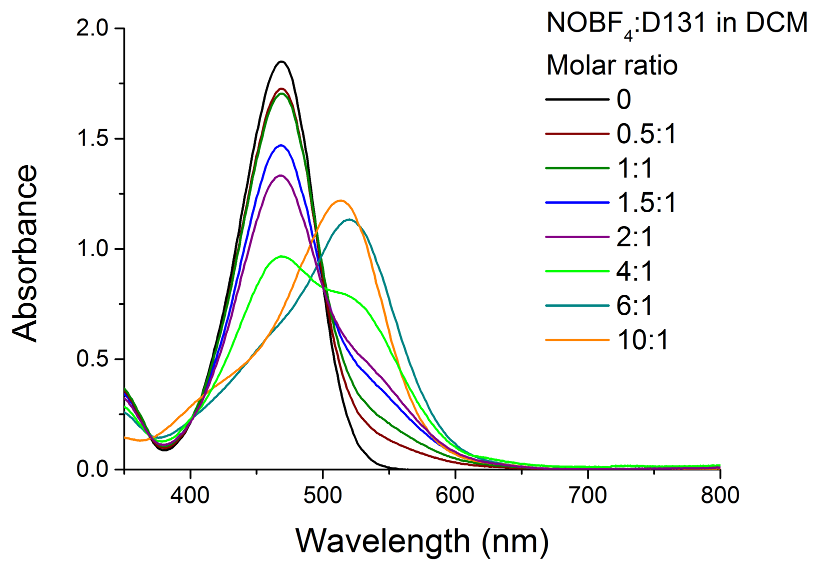


**Supplementary Figure 6:** Absorption spectra of D131 during chemical oxidation with $NOBF_4$ in dichloromethane. The spectra show the emergence of a D131 cation peak around 540 nm. $NOBF_4$ was predissolved in anhydrous acetonitrile. The experiment was carried out in ambient condition. Traces of water in solution could be the reason for the high $NOBF_4$ to D131 molar ratio required to oxidise a substantial fraction of the dye.

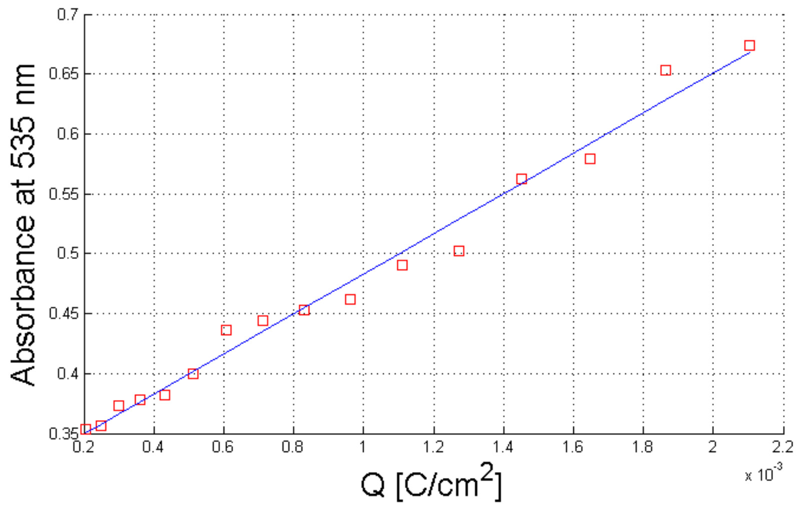


**Supplementary Figure 7:** Spectroelectrochemical data of D131 anchored to $TiO_2$ in 0.1M TBAP in acetonitrile. The graph shows measurements of the film's absorbance at 535 nm plotted against the charge per unit area injected in the film. The extinction coefficient of oxidised D131 was calculated considering the following relation for the slope of the fitted line $dA(535\text{nm}) / dQ = (\varepsilon_{D131+,535nm} - \varepsilon_{D131,535nm})/q$ where $A$ is the absorbance of the sample, $Q$ is the charge per unit geometric area, $\varepsilon_{D131+,535nm}$ and $\varepsilon_{D131,535nm}$ are respectively the extinction coefficient of oxidised and ground state D131 at 535 nm and $q$ is the charge of an electron. $\varepsilon_{D131+,535nm}$ was determined to be 19,400 $M^{-1}$ $cm^{-1}$ this way. UV-visible spectra were taken at 1Hz while also measuring cyclic voltammetry (CV) at a scan rate of 10 mV $s^{-1}$ in a three electrode cell using the sample as working electrode, a platinum counter electrode and a silver wire as quasi reference electrode. For details about the experimental setup, the CV measurement and the UV-visible absorption data see the supporting information of Moia, D. *et al.* [1].

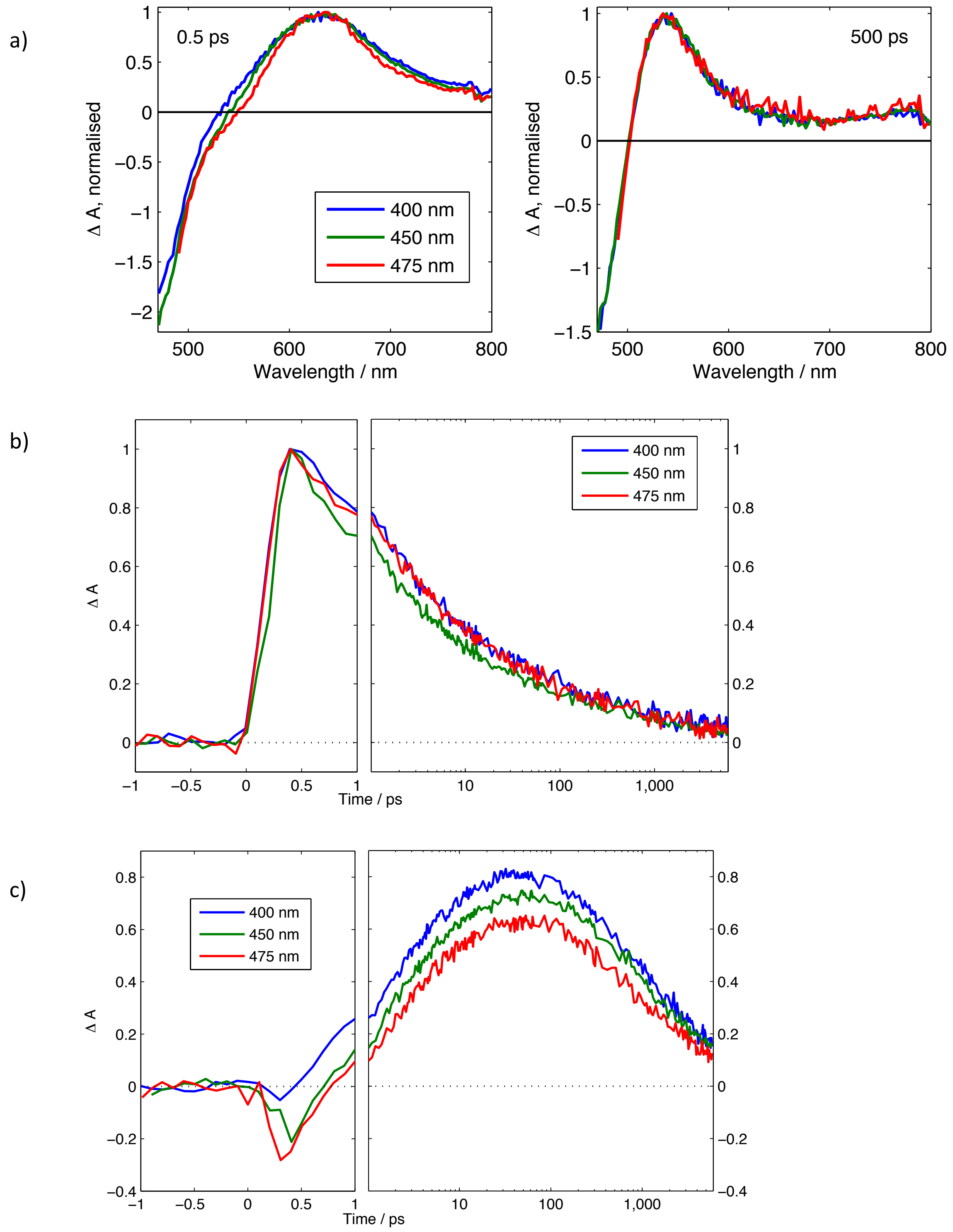


**Supplementary Figure 8:** Excitation wavelength dependence of fsTAS of D131 on $Al_2O_3$ (100% coverage). a) FsTAS spectra measured at 0.5 ps delay time (left) and at 500 ps delay time (right). Spectra at 0.5 ps mostly show the excited state of D131 with small variations in the width of the peak. The spectra at 500 ps show the new state with identical spectra for all excitation wavelengths indicating complete conversion of the excited state to the new state after this time. b) Kinetic traces at a probe wavelength of 640 nm. The traces are normalised to the initial amplitude and show that the decay of the excited state of D131 is very similar at different excitation wavelengths. c) Kinetic

traces at a probe wavelength of 535 nm. The traces are normalised to the initial amplitude at 640 nm and show that the generation of the new state happens for all excitation wavelengths but is somewhat less efficient compared to the initial amount of excited state for longer excitation wavelengths.

a)

b)

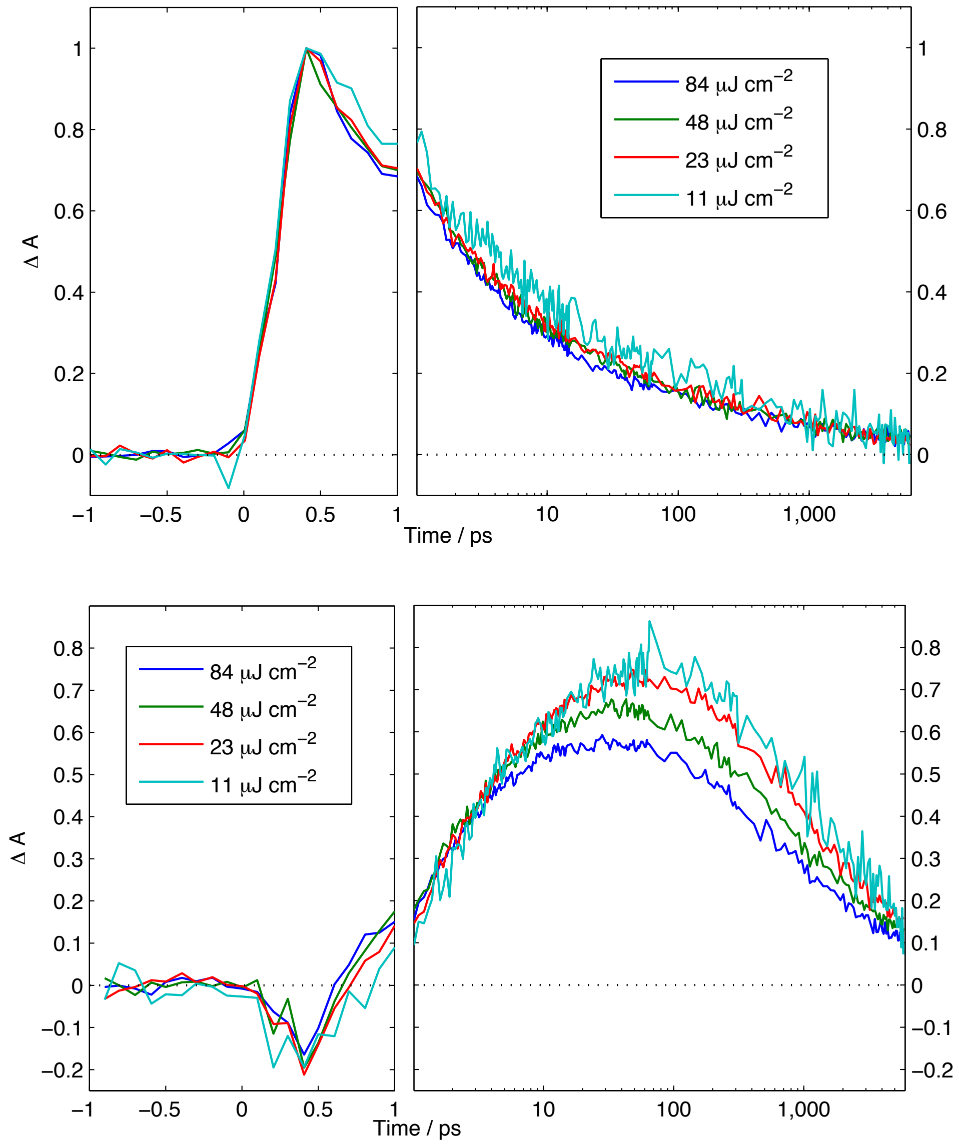


**Supplementary Figure 9:** Power dependence of fsTAS of D131 on $Al_2O_3$ (100% coverage) excited with 450 nm. a) Normalized kinetic traces at a probe wavelength of 640 nm with different intensities showing the D131 excited state kinetics. The traces show only a small dependence on the pump power. b) Kinetic traces at a probe wavelength of 535 nm showing the kinetics of the new state formation. The traces are normalised to the initial amplitude at 640 nm and show that the generation is less efficient at higher laser powers.

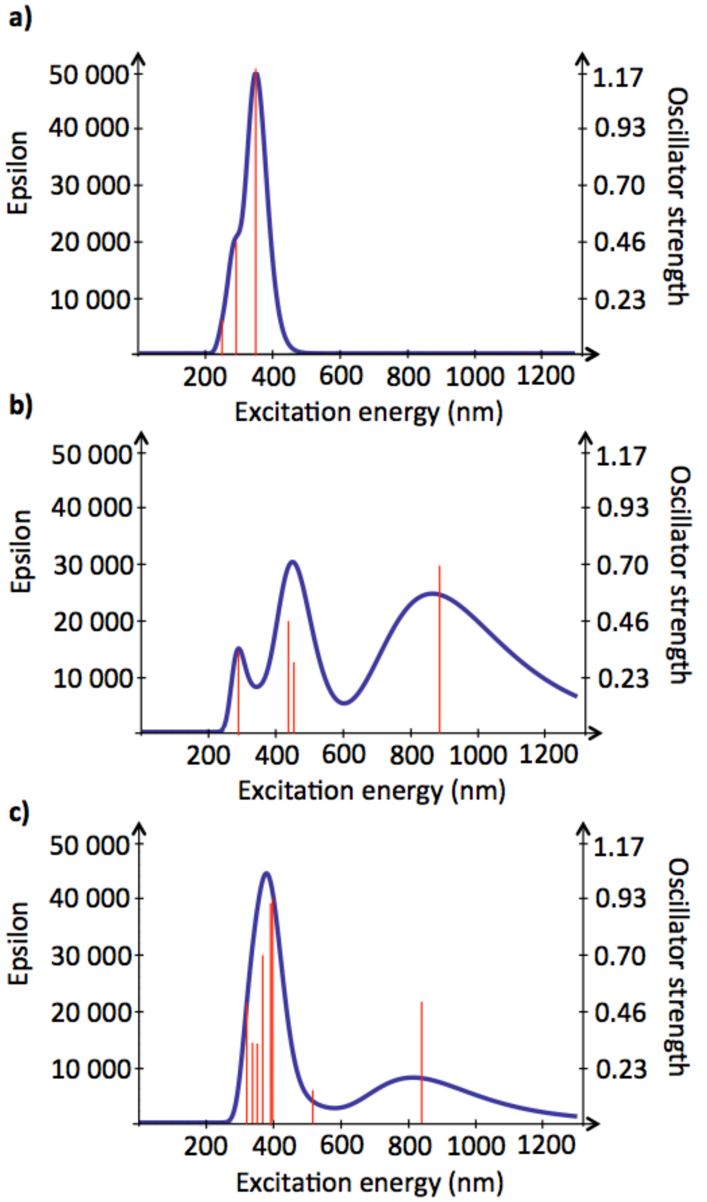


**Supplementary Figure 10:** Calculated absorption spectra of D131 in different oxidation states from TDDFT. a) ground state, b) oxidized, c) reduced. The geometry of D131 was optimized for the three different oxidation states using DFT (Gaussian 09, cam-B3LYP/TZVP-6D). Single point energy calculations were run from these optimized geometries at the TDDFT level (cam-B3LYP/TZVP-6D), giving the spectra plotted. For the ground state and oxidized dye the spectra match well with the experimentally observed absorption spectra.

a) b)

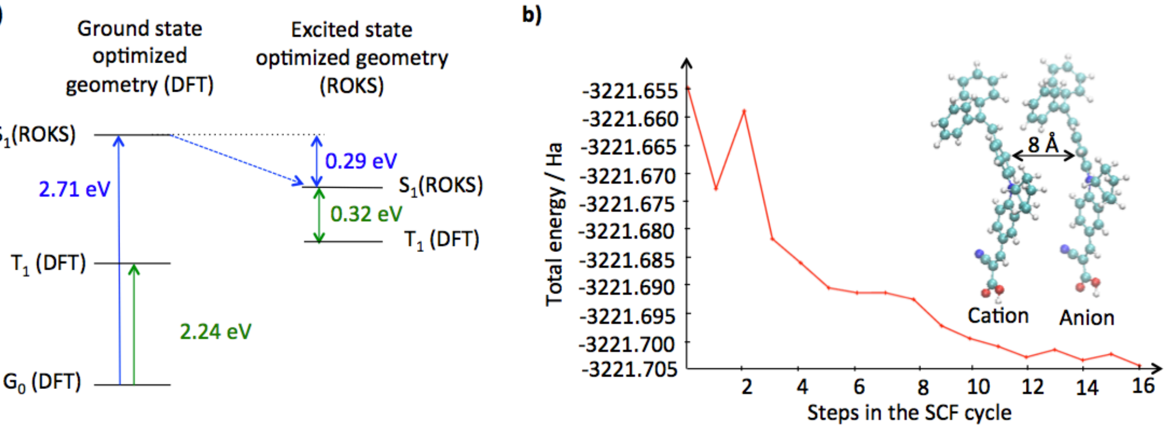


**Supplementary Figure 11:** a) Energy levels of the ground, triplet and singlet excited states of D131 at the optimized geometry of the ground and excited states. All energies were calculated using the Kohn-Sham formalism as implemented in Q-Chem (B3LYP/6-311G(d,p)). Ground and triplet states were simulated with DFT while the singlet excited state calculation was performed with the Restricted Open Kohn Sham (ROKS) method. The vertical energy excitation in the ground state is consistent with experimental observation, as well as the energy difference between the optimized excited and ground states. b) Charge constrained DFT (CDFT) geometry optimization of a pair of D131 molecules whose intermolecular distance is 8A. The overall pair is neutral but the charge of one of the molecule was constrained to be -1 to simulate charge separation across two neighbouring dyes. The CDFT calculation was performed with QChem (B3LYP/6-311G(d,p)). These calculations are notoriously hard to get to converge. Here, we show the variation of the total energy of the pair as a function of the self-consistent field (SCF) cycle steps. Although we do not reach numerical convergence to more than 3 digits, the oscillations starting to appear suggest that it is a good enough approximation to the total energy. Therefore, we use the last energy data point to estimate the energy difference between the reactants and products of the charge separation.

$$\Delta E = E_{CDFT}(D131^{-} + D131^{+}) - E_{G0} - E_{S1}$$

$$\Delta E = (-3221.7043616336 + 1610.9065919313 + 1610.8068515868)\times 27.2 = 0.25\ eV$$

a)

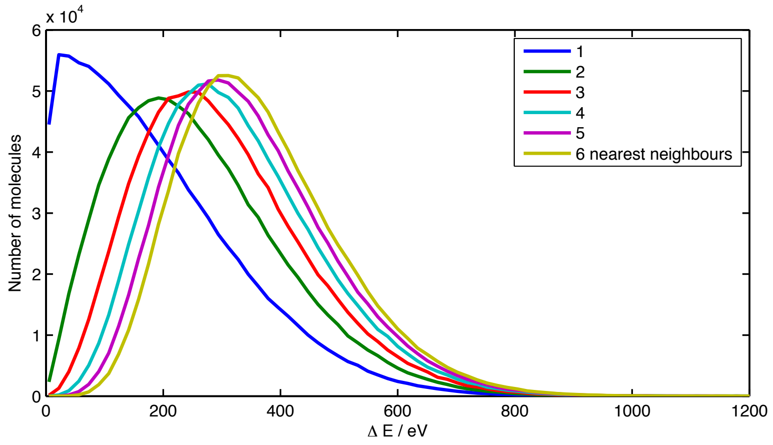

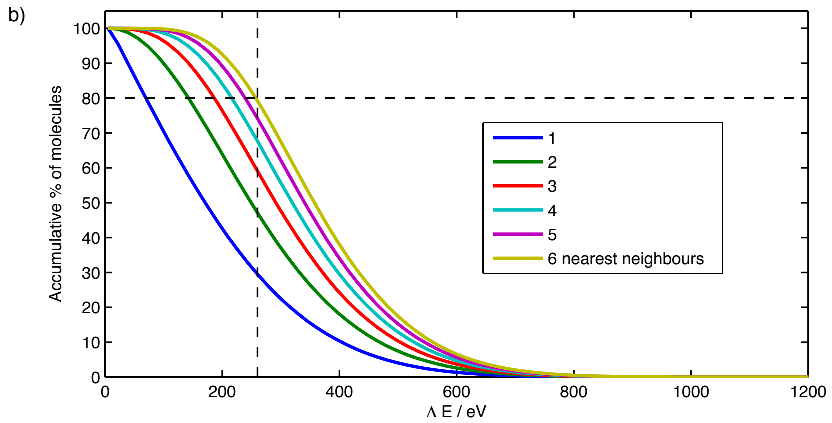


**Supplementary Figure 12:** Largest energy difference ΔE between a molecule and one of its nearest neighbours for an energy variation with a Gaussian distribution and FWHM=0.4 eV calculated using the "normrnd" function in the Statistical Toolbox of Matlab for different numbers of nearest neighbours. a) Number of molecules with an energy difference ΔE out of $10^6$ calculations. b) Accumulative percentage of molecules with an energy separation equal or larger than ΔE.

## S2 Supplementary Tables

**Supplementary Table 1:** Fit parameters for the multi-exponential fitting of kinetics traces of fsTAS measurements. For data at 640 nm, the amplitudes $A_i$ were normalized to add to 1.

| Sample | Coverage | $\lambda_{probe}$ / nm | $A_1$ | $\tau_1$ / ps | $A_2$ | $\tau_2$ / ps | $A_3$ | $\tau_3$ / ps | $A_4$ | $\tau_4$ / ps |
|---|---|---|---|---|---|---|---|---|---|---|
| D131 + $Al_2O_3$ | 100% | 640 | 0.45 | 2.7 | 0.30 | 30.7 | 0.19 | 446 | 0.05 | |
| D131 + $Al_2O_3$ | 100% | 535 | -0.61 | 2.1 | -0.45 | 16 | 0.52 | 880 | 0.44 | 6800 |
| D131 + $Al_2O_3$ | 60% | 640 | 0.38 | 3.0 | 0.31 | 30.5 | 0.25 | 407 | 0.06 | |
| D131 + $Al_2O_3$ | 60% | 535 | -0.17 | 7.5 | -0.24 | 64 | 0.30 | 6700 | | |
| D131 + $Al_2O_3$ | 12% | 640 | 0.21 | 3.2 | 0.25 | 48 | 0.46 | 644 | 0.08 | |
| D131 in polystyrene | | 640 | 0.20 | 2.4 | 0.33 | 58 | 0.45 | 920 | 0.02 | |

**Supplementary Table 2:** Normalized amplitudes, time constants and the average decay times from double exponential fitting of fluorescence decay measurements.

| Sample | Coverage | $\lambda_{probe}$ / nm | $A_1$ | $\tau_1$ / ps | $A_2$ | $\tau_2$ /ps | $t_{av}$ / ps | Err / ps |
|---|---|---|---|---|---|---|---|---|
| D131 + $Al_2O_3$ | 100% | 580 | 0.66 | 74 | 0.34 | 675 | 279 | 2 |
| D131 + $Al_2O_3$ | 60% | 580 | 0.53 | 132 | 0.47 | 1493 | 780 | 20 |
| D131 + $Al_2O_3$ | 12% | 580 | 0.14 | 160 | 0.86 | 1495 | 1310 | 20 |

## Supplementary References:


[1] Moia, D. *et al.* The Role of Hole Transport between Dyes in Solid-State Dye-Sensitized Solar Cells. *J. Phys. Chem. C* 119, 18975–18985 (2015).
